# Why Intracranial Compliance Is Not Utilized as a Common Practical Tool in Clinical Practice

**DOI:** 10.3390/biomedicines11113083

**Published:** 2023-11-17

**Authors:** Seifollah Gholampour

**Affiliations:** Department of Neurological Surgery, University of Chicago, Chicago, IL 60637, USA; seifgholampour@bsd.uchicago.edu

**Keywords:** intracranial compliance (ICC), viscous component, gradual onset brain disorders, brain disorder, neurosurgery, cerebrospinal fluid, brain biomechanics, intracranial pressure (ICP), clinical application, diagnostic tool

## Abstract

Intracranial compliance (ICC) holds significant potential in neuromonitoring, serving as a diagnostic tool and contributing to the evaluation of treatment outcomes. Despite its comprehensive concept, which allows consideration of changes in both volume and intracranial pressure (ICP), ICC monitoring has not yet established itself as a standard component of medical care, unlike ICP monitoring. This review highlighted that the first challenge is the assessment of ICC values, because of the invasive nature of direct *measurement*, the time-consuming aspect of non-invasive *calculation* through computer simulations, and the inability to quantify ICC values in *estimation* methods. Addressing these challenges is crucial, and the development of a rapid, non-invasive computer simulation method could alleviate obstacles in quantifying ICC. Additionally, this review indicated the second challenge in the clinical application of ICC, which involves the dynamic and time-dependent nature of ICC. This was considered by introducing the concept of time elapsed (TE) in measuring the *changes* in volume or ICP in the ICC equation (volume change/ICP change). The choice of TE, whether short or long, directly influences the ICC values that must be considered in the clinical application of the ICC. Compensatory responses of the brain exhibit non-monotonic and variable changes in long TE assessments for certain disorders, contrasting with the mono-exponential pattern observed in short TE assessments. Furthermore, the recovery behavior of the brain undergoes changes during the treatment process of various brain disorders when exposed to short and long TE conditions. The review also highlighted differences in ICC values across brain disorders with various strain rates and loading durations on the brain, further emphasizing the dynamic nature of ICC for clinical application. The insight provided in this review may prove valuable to professionals in neurocritical care, neurology, and neurosurgery for standardizing ICC monitoring in practical application related to the diagnosis and evaluation of treatment outcomes in brain disorders.

## 1. Introduction

Brain disorders affect 6.75% of the American adult population [1] and constitute a significant cause of morbidity worldwide, with their incidence steadily increasing [2]. Clinical overlaps and similarities in medical imaging can lead to misdiagnosis between various brain disorders, such as normal pressure hydrocephalus (NPH) and Parkinson’s or Alzheimer’s disease [3,4]. The prediction of treatment outcomes for brain disorders can also be challenging, even in patients with identical clinical conditions [5,6,7,8]. These complexities may be related to the lack of clear and comprehensive knowledge concerning the mechanisms behind brain disorders. Brain tissue is a complex biomaterial with a highly heterogeneous and anisotropic microstructure, variable biomechanical properties, and dynamic behavior that can change in response to different loadings due to various brain disorders [7,9,10]. Besides other indicators utilized to evaluate brain disorders, intracranial compliance (ICC) has also been employed to complement the interpretation of intracranial pressure (ICP) in neurocritical care and to aid in predicting the deterioration of brain function [11]. ICC is the volume-buffering capacity representing cranial adaptation and CSF–brain interaction. In other words, ICC is the ability of the intracranial system to adjust its volume to maintain stable ICP. Maintaining stable ICP is crucial for the stability of the cerebral blood flow system, preventing tissue damage, and ensuring optimal neural activity [12]. Previous studies showed that ICC is a critical parameter to understanding the mechanisms underlying some brain disorders and the brain’s responses to various pathological processes [13]. Foreman et al. demonstrated how multimodality monitoring, which could include ICC monitoring, provides data to guide the precision management of patients with brain disorders, such as traumatic brain injury [14]. Rabai et al. also emphasized that understanding the changes in ICC could enhance the effectiveness of intraoperative neuromonitoring, which is important for the near real time assessment of neuronal pathways during surgery [15]. ICC can also be useful in predicting clinical outcomes in some brain disorders, such as hydrocephalus, cerebral atrophy, intracranial hypertension, traumatic brain injury, and intracranial hematoma. Therefore, ICC can play a prominent role in advancing our conceptual understanding of the pathophysiology of certain brain disorders, while also serving as a valuable tool in the diagnosis and evaluation of treatment outcomes in a broad range of brain disorders [16].

The basis for ICC measurement was first suggested 70 years ago in an animal study [17]. Subsequent research has consistently emphasized the clinical significance, efficacy, and sensitivity of ICC in diagnosing and evaluating treatment outcomes across a spectrum of brain disorders. Despite significant advances in medicine broadening the scope of neurosurgical practice [18], the practical implementation of ICC in clinical settings continues to pose intricate challenges. While ICP stands as a well-established metric for investigating a wide array of brain disorders, ICC, a more comprehensive concept that encompasses ICP, is not commonly employed as a clinical indicator by clinicians and it is typically utilized in research projects. This review is dedicated to addressing these concerns, discussing the challenges, and outlining the difficulties associated with the practical application of ICC. It aims to provide insight and guidance for neurosurgeons, neurologists, and professionals in neurocritical care who play a pivotal role in neuromonitoring during the evaluation of brain disorders.

## 2. Challenges in Measurement, Calculation, and Estimation of ICC

**Figure 1:**
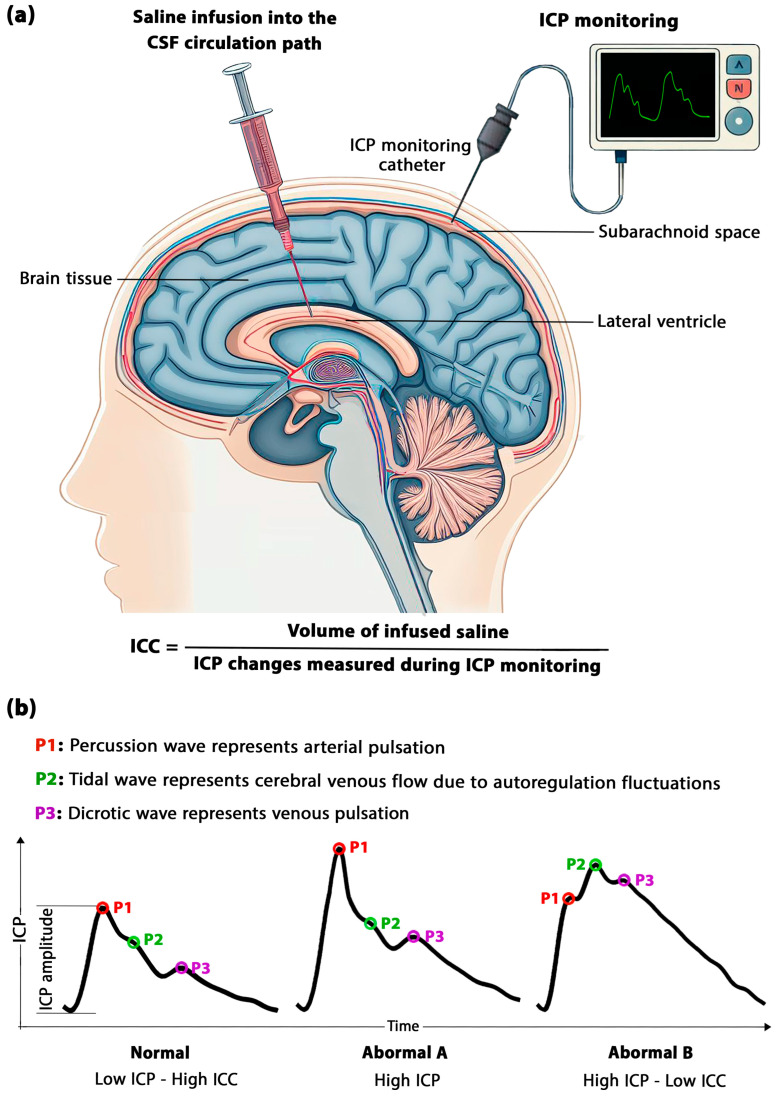
(**a**) Direct ICC Measurement: A schematic illustrating ICC measurement. For clarity, red color is used to represent the infused saline; in actual conditions, it is colorless, resembling CSF. The injection location in this figure is in the lateral ventricle, but it can also be in the subarachnoid space or lumbar space. (**b**) This panel displays ICP waveforms over time to estimate ICC based on ICP waveform morphology. The normal ICP waveform is synchronous with the arterial pulse. Abnormal A: In the early compensation stage, there is an increase in the peak interval between P1 and P2, signifying an increase in cerebral vasculature pulsations and ICC. Abnormal B: When ICP remains consistently high, this initial reaction is followed by a gradual decrease in the peak interval between P1 and P2. CSF: Cerebrospinal fluid; ICP: Intracranial pressure; ICC: Intracranial compliance.

The primary techniques for the direct *measurement* of ICC involve monitoring changes in cerebrospinal fluid (CSF) volume within the craniospinal space [19]. These techniques are generally based on the Marmarou method, which involves infusing a known volume of saline into the lumbar or cranial area and measuring the resulting increase in pressure (Figure 1a). The ratio of the injected volume to the increase in ICP measures the ICC. The main limitation of these techniques was their invasiveness, posing a risk of complication [20]. Moreover, in specific clinical scenarios, even a minor alteration in CSF volume can lead to changes in ICP, resulting in unpredictable clinical consequences. Similarly, Okon et al. revealed that techniques involving the active injection of excessive fluid into the CSF circulation system might not be suitable in cases of elevated ICP [21]. Smielewski et al. demonstrated that these techniques, including constant infusion, constant pressure infusion, bolus manipulation, and lumbar ventricular perfusion, could introduce potential errors in pressure readings due to possible vasomotor responses [22]. Our recent study also confirmed the potential for methodological heterogeneity in ICC values measured using these methods [23]. Hence, there was a need to explore alternatives for measuring ICC without adding or removing fluid from the craniospinal space which might involve manipulating the natural CSF circulation system and non-invasive methods emerged as superior options.

Alperin et al. initiated efforts to *calculate* ICC non-invasively through computer simulations [24,25]. We developed and optimized these non-invasive ICC calculations, employing fluid-structure interaction (FSI) computer simulations to enhance calculation accuracy [26,27,28,29]. Additionally, we designed and fabricated an in vitro model for experimental ICC measurements [30]. Despite these advancements, it is noteworthy that computer simulation methods can be time-consuming and may not be feasible in emergency conditions. Furthermore, the translation of in vitro measured ICC for human application is not fully facilitated. Hence, several studies have endeavored to gauge ICC non-invasively, although they were unable to directly measure or calculate ICC values. Instead, they attempted to *estimate* ICC effects indirectly based on measurable parameters, such as optic nerve sheath diameter [31], transcranial Doppler ultrasound [32], ICP wave amplitude [33], blood pulse analysis [34], and bioelectrical models [35]. One of the most well-known approaches in the estimation methods is the prediction of changes in ICC based on ICP waveform morphology (Figure 1b). Although estimation methods may only approximate the general trend of ICC changes, they may be a more feasible option in neurological emergency situations compared to the relatively slower pace of computer simulations.

## 3. Challenges in the Definition and Concept of ICC

### 3.1. Time-Dependent Viscous Component of the Brain

Comprehending and defining the appropriate brain model is one of the most important priorities that should be clarified for a conceptual understanding of ICC. The brain is composed of different substructures, including white and gray matter, blood vessels, neurons, and fluids such as extracellular fluid. Previous studies modeled the brain as a poroelastic structure based on brain consolidation theory [36]. The brain poroelastic model assumes the brain to be a porous medium consisting of solid and fluid phases, describing the time-dependent interactions between these phases in the brain. On the other hand, brain substructures have different time-dependent viscous properties that contribute to the brain’s overall damping characteristics. Previous studies confirmed that a time-dependent viscous component must be considered in the definition of the brain model [37]. Hence, in addition to the importance of the scale of strain (short or long) in the brain model, the time-dependent viscous component is also necessary to achieve agreement between mathematical biphasic characteristics and experimental material properties of the brain [38]. Hrapko et al. used a large strain viscoelastic framework to define one of the most prominent time-dependent stress–strain models for the brain [39]. In addition to poroelastic models, various studies have also tried to consider a time-dependent viscous component in the brain model under a viscoelastic, hyper-viscoelastic, or poro-hyperviscoelastic model (Table 1). Gholampour et al. and Cheng et al. used a poro-viscoelastic model to define the *most appropriate* constitutive model for the brain [26,28,40]. On the other hand, Elkin et al. showed that the best conformity with experimental data is obtained when the viscoelastic component of the brain is fitted to the shear modulus ($Gr\left(t\right)$) using the Prony series [41]. Hence, we applied this method to consider the parameter of time (*t*) in our poro-viscoelastic brain model using Equation (1) [26,28]:

(1) $$Gr\left(t\right)=G_{0}(1-\sum_{k=1}^{N}g_{k}^{p}{(1-e}^{-\left(\frac{t}{\tau_{k}}\right)}))$$

where $G_{0}$, $g_{k}^{p}$, and τ*_k_* in Equation (1) are input shear modulus, relaxation modulus, and relaxation time, respectively. It can be deduced that, regarding the proven impact of the viscous component in the mathematical and computational models of the brain, the role of time and the time-dependency of the brain are irrefutable in the study of brain function and, consequently, in understanding ICC in brain disorders.

### 3.2. The Role of Time in the Formulation of ICC

**Figure 2:**
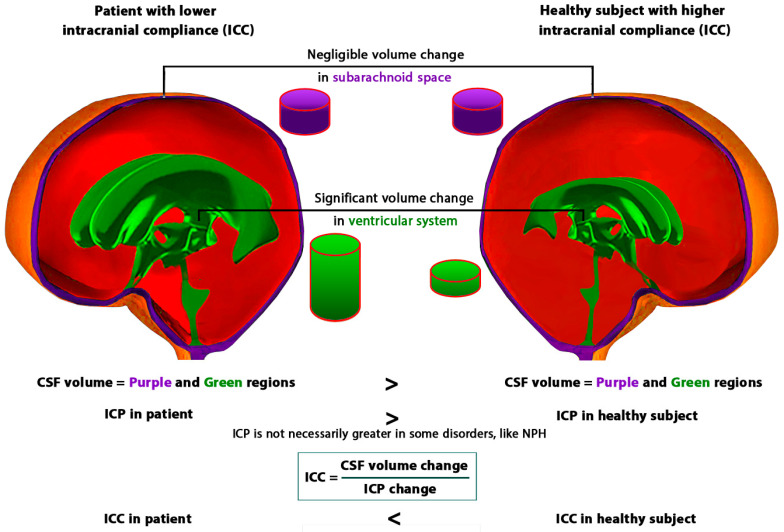
Comparison of ICC concepts based on Equation (2) in a patient and a healthy subject. It should be noted that our previous studies indicated negligible volume change in the subarachnoid space, with the main volume change occurring in the ventricular system [26,28], as indicated in this figure. CSF: Cerebrospinal fluid; ICP: Intracranial pressure; ICC: Intracranial compliance.

The measurement or calculation of ICC values in previous methods was typically based on the classic equation of ICC, as illustrated in Figure 2, defined by the change in volume divided by the change in ICP (Equation (2)). The industrial materials, except for specific types of composites, are non-viscous and their behavior is independent of time. Hence, the parameters used in the ICC equation (changes in volume and pressure) have adequate potential to define compliance in these materials. Nevertheless, the brain models demonstrated that the physiological and biomechanical functions of the brain exhibit time-dependency, as discussed in Section 3.1. Our previous study also showed the importance of the creep behavior (time-dependent deformation) of brain tissue in the treatment process of some brain disorders, such as hydrocephalus [27]. Furthermore, some brain disorders are gradual-onset, which means they develop slowly, and their onset and progression occur over time. For instance, NPH, primary intracranial hypertension, and primary hydrocephalus frequently manifest gradually, with many patients experiencing elevated ICP over extended periods [26,55]. Additionally, cognitive disorders such as Alzheimer’s disease and Parkinson’s disease are also characterized by a gradual onset, where symptoms progressively emerge over time. Previous studies have also proved time-dependency and long-term alterations in CSF dynamics and brain morphometric parameters in patients with CSF disorders [26,29,49,59]. Therefore, in addition to the defined parameters in the ICC equation, ‘time’ may also play a significant role in the formulation and, consequently, in the measurement or calculation of ICC. Certain previous studies formulated compliance based on pressures and volumes of cerebral blood and CSF [60,61,62]. Some studies defined ICC based on the elastic modulus of brain tissue, ICP, and intracranial volume [63,64,65]. Additionally, others formulated ICC based on the dynamics of injected CSF, ICP, and resistance to CSF outflow in invasive direct ICC measurement methods [66]. However, these studies have not directly considered the parameter of time in their ICC equations. We considered that the ICC equation can remain in its general form (Equation (2)) and consider the effect of time in the concept of ‘change’. ‘Change’ of volume or ICP in the ICC equation (Equation (2)) means *V*_2_ − *V*_1_ or *ICP*_2_ − *ICP*_1_ [55]. It can be deduced that we can consider the time elapsed (TE) between the measurements of *V*_2_ and *V*_1_, or *ICP*_2_ and *ICP*_1_ as representative of the parameter of time in the *ICC* equation:

(2) $$ICC=\frac{Volume\;change\;(∆V)}{Intracranial\;pressure\;change\;(∆\;ICP)}=\frac{V_{2}-V_{1}}{{ICP}_{2}-{ICP}_{1}}$$

### 3.3. Approaches to TE in ICC Assessment

Table 2 illustrates variations in ICC values across different assessment methods and various TE values. The presence of these significant differences, as reflected in inconsistent ICC results, can offer insight into why, despite the clinical importance of ICC, its monitoring has not yet become a standard component in clinical application [67]. Table 2 and previous studies [26,30] also indicated that the values of TE in previous non-invasive ICC measurement methods (in vitro, lumped model, and computer simulation) were less than one minute. The corresponding values for TE in previous invasive ICC measurement methods (bolus injection and lumbar or ventricular infusion) had different values (Table 2). These differences may be attributed to variations in the clinical conditions of the patients and the different time taken to reach plateau ICP during the test. Hence, a question will be raised whether these TEs used in previous non-invasive and invasive ICC assessments are sufficiently long to demonstrate the complete effect of the brain’s time-dependent viscous component. This component, reflecting the brain’s load-history-dependent behavior, pertains to its ability to dissipate loads on the brain in various disorders while also resisting deformation. Therefore, considering the influence of the viscous component is essential in ICC assessment to ensure its correct application in practical scenarios. Recently, we demonstrated that before two months, the complete effect of the viscous component of the brain had not yet appeared in the hydrocephalic brain [27,55]. This means that the appropriate TE for measuring ICC in patients with primary hydrocephalus should not be short and should be at least two months. Evaluating the brains of healthy rats subjected to loading due to electrode implantation also revealed that the effects of loading did not completely dissipate within two months [68]. Moreover, brain material properties, including shear and elastic modules, did not stabilize during this period, reflecting the time-dependent nature of the ICC concept. Similarly, Boulet et al. obtained comparable results, confirming significant changes in brain material properties in rat brains over a month-long period [69]. Therefore, we can categorize the evaluation of ICC into two approaches: ICC in a short TE and ICC in a long TE [55]. 

Comparing ICC behavior using these two different approaches can be helpful in clarifying certain complexities related to the clinical application of ICC for some brain disorders. For example, Eide and Brean showed when the ICC decreases in NPH patients in a short TE, the brain material behaves similarly to a linear elastic [78]. While, another study showed that the changes in elastance, stiffness, and creep of the brain in a long TE were non-linear and non-monotonic in patients with primary communicating hydrocephalus [27]. Therefore, the correct choice of TE, whether short or long, directly influences the attainment of stable brain material properties and also affects the manifestation of the impact of the brain’s viscous component, which is of great importance in the clinical application of the ICC indicator.

## 4. Discussion

The complex and dynamic behavior of the brain can change in response to various loadings due to different brain disorders, and ICC plays a crucial role in understanding the mechanisms and complexities underlying these changes. Neuromonitoring can also benefit significantly from the involvement of ICC, which not only serves as a diagnostic tool but also has the potential to improve patient outcomes in neurocritical care. As a result, the challenges associated with the practical application of ICC for clinical purposes hold significant importance in the context of brain disorders. This review is expressly crafted to meticulously address these challenges.

One of the challenges we identified is the absence of a standardized and appropriate method for ICC assessment. Direct ICC measurement techniques, being invasive and not easily performed, pose a risk to patients and may, at times, lack the required accuracy. However, non-invasive, computer simulation methods used to calculate ICC are time-consuming and may not be feasible in emergency situations. Estimation methods, on the other hand, cannot quantify the ICC value; instead, they can only monitor the general trend of ICC changes. Furthermore, most of these estimation methods rely on the correlation of ICC with available parameters. These statistical correlations are not always correct in all conditions, leading to potential inaccuracies in ICC evaluation. Therefore, the development of a non-invasive, accurate, and quick method for measuring ICC remains a significant challenge for the practical use of ICC in neurology and neurosurgery. The second challenge is linked to the definition of the ICC concept, given its time-dependent nature. In this context, TE has been defined as a representation of ICC time-dependency within the ICC equation. Our review has brought to light that the choice of TE, whether short or long, directly influences the manifestation of the brain’s viscous component and reflects the dynamic nature of ICC in clinical approaches. Therefore, in addition to the first challenge related to the assessment of ICC values, the definition of ICC poses another significant challenge, as the accuracy of ICC assessment is notably affected by the selection of TE, whether short or long.

**Figure 3:**
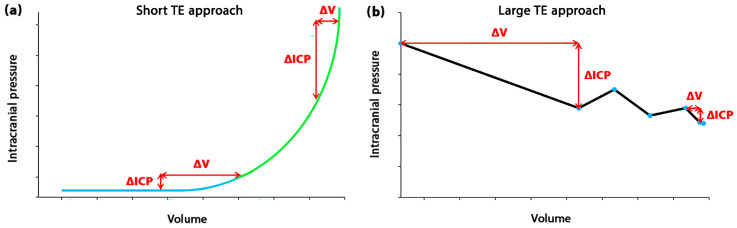
Volume-ICP relationships in short and long TEs: (**a**) shows the monotonic trend of the volume-ICP (VI) curve in a short TE. Two compensatory reserve zones are shown. The first zone is the upper reserve zone (blue line). In this zone, the ICP remains relatively stable despite changes in volume. This is due to the brain’s ability to compensate by reducing the volume of CSF and increasing blood flow out of the brain. Another zone is the lower reserve zone (green line). In this zone, the brain’s compensatory mechanisms are exhausted, and further increases in volume lead to a rapid increase in ICP to reach a plateau ICP; (**b**) shows the non-monotonic trend of the volume-ICP curve in a long TE after treatment for a hydrocephalus patient. The compensatory response of the brain could somewhat recover at certain times in a long TE. This curve is not divisible into some specific compensatory reserve zones. ICP: Intracranial pressure, ICC: Intracranial compliance, TE: Time elapsed, CSF: Cerebrospinal fluid.

**Figure 4:**
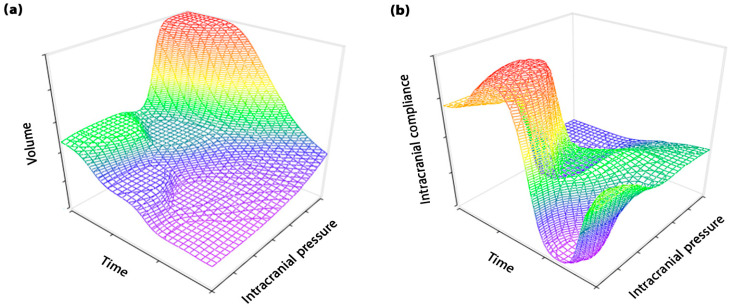
Changes in compensatory response of the brain in a long TE: (**a**) shows the changes in volume and ICP at different time points in a long TE. This shows that the parameter of time, in addition to volume and ICP, can affect the compensatory response of the brain in a long TE—contrasting with short TE; (**b**) shows changes in ICC with ICP at different time points. This shows that the ICC trend and the compensatory response of the brain can have non-monotonic and variable changes in a long TE. ICP: Intracranial pressure, ICC: Intracranial compliance, TE: Time elapsed.

In light of the challenges in the assessment of ICC value and the dynamic nature of ICC behavior discussed above, it becomes imperative to delve further into the analysis of the impact of TE approaches on the changes in the volume-ICP curve (VI curve), as ICC is the slope of this curve (Equation (2)). This in-depth exploration will provide a nuanced understanding of the ICC concept and its changes in various brain disorders, laying a foundation for the practical application of ICC in clinical settings. The VI curve plays a crucial role in understanding the compensatory response of the brain in different brain disorders, providing valuable insight into the intricate dynamics of brain functions. Previous studies strongly believed the trend of the VI curve to be monotonic over short TE in patients with different types of brain disorders (Figure 3a) [24,79,80]. However, Okon et al. expressed doubt about the monotonic trend of VI curves in the short TE [21]. They showed that the trend of this curve in patients with idiopathic intracranial hypertension was not necessarily monotonic. Our evaluations showed that the trend of this curve is non-monotonic in hydrocephalus patients in a long TE (fifteen months after treatment) (Figure 3b and Figure 4a) [27,55]. It can also be understood from a study by Tisell et al. that there is a non-monotonic trend in the VI curve over a long TE (three months after treatment) in hydrocephalus patients [81]. In addition to the variable trend of the VI curve and the compensatory response of the brain in short and long TE, we also showed that in a long TE, ICC does not necessarily increase with decreasing ICP levels, and the ICC oscillatory increases during the treatment process (Figure 4b) [27,55]. This notable finding should also be taken into account in the clinical application of ICC.

Previous studies showed descents in the VI curve at certain times in a long TE for some disorders like hydrocephalus (Figure 3b) because of the non-monotonic trend of this curve [27,55]. Similar descents were also demonstrable in a study by Tisell et al. [81]. This means in the context of ICC assessment using a long TE approach, ICC values and, consequently, the compensatory response of brain tissue may partially recover at specific times. It is worth mentioning that when the brain is in an uncompensated status in a short TE, it is traditionally defined as a high-risk condition [67]. However, the temporary noncompensatory responses of the brain caused by the non-monotonic trend of the VI curve in a long TE cannot necessarily be deduced as a high-risk condition in the practical application of the ICC indicator. In addition, two separate compensatory reserve zones were shown in the VI curve in a short TE (Figure 3a: blue and green colors). The ICC values and pulse amplitude of ICP change in these two zones. However, the VI curve in a long TE was not divisible into specific, meaningful compensatory reserve zones in gradual-onset brain disorders, such as hydrocephalus (Figure 3b). Overall, the general trend (mono-exponential) and compensatory reserve zones (two zones) of the VI curve in a short TE were approximately the same in different types of brain disorders, such as hydrocephalus [24,82], brain edema [79], and brain injury [80], and they were different from a long TE approach.

The correct choice of TE (short or long) depends on the strain rate and loading time in that particular brain disorder under assessment for ICC. Strain rate is a measure of how quickly the brain is being deformed under loading caused by a brain disorder. The loading time refers to the duration of the loading that is applied to the brain in a specific brain disorder, and it is a measure of how long the brain is being subjected to loading. The strain rate and loading time vary across different types of brain disorders. For instance, these parameters significantly differ in traumatic brain injury and some gradual-onset brain disorders, such as hydrocephalus. Therefore, it can be inferred that the practical utilization of ICC also depends on the strain rate and loading duration specific to each individual brain disorder, taking into account the two approaches used in ICC assessment. In addition, to measure or calculate ICC, it is essential to determine two statuses, denoted as 1 and 2 in Equation (2), which are required for computing the ‘change’ in volume and ICP. In emergency conditions of certain brain disorders, even primary hydrocephalus, known for its substantial strain rate and loading duration, makes the application of ICC in a long TE approach unfeasible due to the time required to establish these two statuses for the ICC equation. In such scenarios, ICC estimation methods may prove more useful. Conversely, in cases like NPH, ICC can be exclusively used for diagnosis and evaluating treatment outcomes. This is because the conditions of NPH patients are not always emergencies. NPH is generally a gradual-onset brain disorder and other available indicators, such as ICP, gait analysis, or cognitive examinations, may not always be effective in evaluating these diseases [23,83]. However, a standardized numerical threshold for ICC values to differentiate between healthy subjects and patients is yet to be established, even for conditions such as NPH or in non-emergency situations, for the practical implementation of ICC as a clinical indicator. Given the inherent capability of computer simulation as the sole method capable of non-invasively quantifying ICC values, the development of novel rapid simulations in future studies [84] holds promise for the practical application of ICC in these disorders. This approach can define a threshold level that distinguishes ICC values between healthy subjects and patients.

The recovery behavior of the brain during the treatment process (unloading condition) is of great importance in the management of brain disorders [27]. Previous studies have demonstrated that the biomechanical response of the brain in loading (i.e., afflicted with a brain disorder) and unloading (i.e., during some treatment processes) conditions is different [85]. Elastic hysteresis behavior of the brain is one of the primary reasons for this difference, specifically in disorders caused by *cyclic* loading, i.e., in hydrocephalus and intracranial hypertension that are caused by *pulsatile* elevated ICP. The theory of elastic hysteresis of the brain states that the brain stores energy during loading; however, not all of this energy is released during unloading, as some of it is dissipated due to internal friction. This leads to a residual (permanent) deformation in brain tissue after the treatment process. The differences in residual deformation of the hydrocephalic brain were indicated in short and long TEs [27,38]. This theory, which is provided by Lesniak et al., confirmed the direct impact of elastic hysteresis behavior of the brain on ICC changes [86]. Comparing ICC values in loading and unloading conditions over a long TE in future studies can be useful for developing the theory of elastic hysteresis. This comparison can further clarify how ICC changes in loading and unloading conditions in a long TE, potentially enhancing the practical utility of ICC, especially in gradual-onset brain disorders. In addition, previous studies showed a decrease in glymphatic drainage and ICC value in a short TE for patients with NPH, traumatic brain injury, and Alzheimer’s disease [87,88,89]. Evaluation of the changes in ICC and glymphatic drainage in a long TE in future studies may be useful to support the hypothesis of glymphatic dysfunction as the underlying pathophysiology of gradual-onset brain disorders. On the other hand, wireless, batteryless, and minimally invasive implantable sensors for ICP monitoring have been introduced in recent years [90]. These sensors consist of ultrathin, flexible spiral coils connected in parallel to a capacitive microelectromechanical systems pressure sensor. Despite their potential, a careful and thorough practical validation of their efficiency, accuracy, and reliability has not been conducted. Future studies can further develop these methods and validate their results using other clinically standard ICP measurement approaches to address the shortcomings of previous methods in ICC monitoring. As the volume change in Equation (2) for ICC measurement can be assessed non-invasively using imaging data, the primary challenge in the clinical application of non-invasive ICC monitoring lies in ICP measurement, for which these methods offer potential solutions.

## 5. Conclusions

ICC holds significant potential as a pivotal clinical indicator for diagnosing and evaluating the treatment outcomes of brain disorders, while also exerting a substantial influence on the efficacy of neuromonitoring techniques. However, unlike ICP—a well-established practical metric for studying a diverse range of brain disorders—ICC is not commonly employed as a practical tool in clinical approaches. This review highlighted a prominent challenge in the practical application of ICC—the absence of a standardized and appropriate assessment method. Recognizing that computer simulations are the only method capable of non-invasively quantifying ICC values, future studies focusing on the development of rapid simulations hold promise in mitigating the challenges associated with the practical application of ICC in clinical contexts. Another significant challenge identified in the clinical use of ICC was its dynamic nature and the time-dependent concept of ICC. Differences in ICC values observed over long TE and short TE approaches in specific brain disorders emphasize the importance of selecting the appropriate TE, a factor that should be taken into account in the clinical application of ICC. The review shed light on the reasons and challenges contributing to the lack of standardization in ICC monitoring for practical application in the diagnosis and evaluation of treatment outcomes of brain disorders. The findings of this review also held practical value for healthcare professionals in neurocritical care, neurologists, and neurosurgeons, offering insight that can contribute to the refinement of neuromonitoring strategies, enabling more precise interventions at critical moments.

## Figures and Tables

**Table 1:** Different time-dependent brain models.

Brain Model	Authors	Type of Brain Disorder	Solving Method	Brain Regions	Source
Poroelastic	Yuan et al.	Healthy subjects under drug infusion	Mathematical analysis based on arbitrary Lagrange–Eulerian equations	White matter	[42]
	Lambride et al.	Brain injury	Finite element method	Single region	[43]
	Guo et al.	Alzheimer’s disease	Finite element method	White matter	[44]
	Gholampour et al.	Non-communicating hydrocephalus	Fluid-structure interaction	Single region	[45,46]
Viscoelastic	Li et al.	Healthy subject	Finite element method	Grey and white matter	[47]
	Siegkas et al.	Brain injury	Finite element method	Single region	[48]
	Gholampour et al.	Hydrocephalus	Fluid-structure interaction	Single region	[29,49]
	Harpko et al.	Healthy subject	Mathematical analysis	White matter	[39]
Hyper-visco-elastic	Menghani et al.	Head impact	Finite element method	Basal ganglia, cerebral hemispheres, and corpus callosum	[50]
	Wang et al.	Brain injury	Finite element method	Grey matter, white matter, and pia mater	[51]
	Wilkie et al.	Hydrocephalus	Mathematical analysis using fractional Zener model	Single region	[52]
	Dutta-Roy et al.	Normal pressure hydrocephalus	Finite element method	Single region	[53]
Poro-visco-elastic	Gholampour et al.	Communicating hydrocephalus	Fluid-structure interaction	Single region	[27,54,55]
	Pavan et al.	Brain injury	Finite element method	One region	[56]
	Gholampour	Non-communicating hydrocephalus	Fluid-structure interaction	Single region	[26]
	Cheng et al.	Non-communicating hydrocephalus	Finite element method	White matter	[40]
Poro-hyper-viscoelastic	Hosseini-Farid et al.	Healthy subject	Finite element method	Grey and white matter	[57]
	Forte et al.	Healthy subject	Finite element method	Grey and white matter	[58]

**Table 2:** Comparison of differences in values and assessment methods of ICC, as well as the time elapsed (TE), in hydrocephalus patients. These studies are related to short TE assessments.

Age	Type of Hydrocephalus	Authors	Intracranial Compliance Assessment Method	Procedure Type	Intracranial Compliance (mL/mmHg)	Time Elapsed (Minute)	Source
Adult	Noncommunicating hydrocephalus	Gholampour et al.	Computer simulation	Non-invasive	0.78	0.17	[30]
		Eide	Ventricular constant-flow infusion	Invasive	0.60	15.5	[70]
	Normal pressure hydrocephalus	Kazmierska et al.	Computer-assisted constant-flow infusion	Invasive	0.27	13.2	[64]
		Mase et al.	Computer simulation	Non-invasive	0.003	<1	[71]
		Meier and Bartels	Computer-assisted constant-flow intrathecal infusion	Invasive	0.36	10.5	[72]
		Sahuquillo et al.	Bolus injection, Lumbar and ventricular constant-flow infusion	Invasive	0.33	15.0	[73]
	Communicating hydrocephalus	Eide	Ventricular constant-flow infusion	Invasive	0.66	15.5	[70]
	Hydrocephalus	Lokossou et al.	Lumbar constant-flow infusion	Invasive	0.23	---	[74]
		Eide	Ventricular constant-flow infusion	Invasive	0.6	15.5	[75]
Pediatric	Noncommunicating hydrocephalus	Czosnyka et al.	Computer-assisted lumbar infusion	Invasive	1.27	6.3	[76]
	Acute hydrocephalus	Czosnyka et al.	Computer-assisted lumbar infusion	Invasive	0.97	6.3	[76]
	Hydrocephalus	Shapiro and Fried	Bolus withdrawal and injection	Invasive	0.32	---	[77]
